# Data-driven honeybee antennal lobe model demonstrates how stimulus-onset asynchrony can aid odor segregation

**DOI:** 10.1186/1471-2202-14-S1-P378

**Published:** 2013-07-08

**Authors:** Thomas Nowotny, C Giovanni Galizia, Paul Szyszka

**Affiliations:** 1 School of Engineering and Informatics, University of Sussex, Brighton, BN2 4RQ, UK; 2 Fachbereich Biologie, Universität Konstanz, 78457 Konstanz, Germany

## 

Insects have a remarkable ability to identify and track odor sources in multi-odor backgrounds. Recent behavioral experiments show that this ability relies on detecting millisecond stimulus asynchronies between odors that originate from different sources [1]. Honeybees, *Apis mellifera*, are able to distinguish mixtures where both odors arrive at the same time (synchronous mixtures) from those where odor onsets are staggered (asynchronous mixtures). Surprisingly, this ability persists down to an onset delay of only 6 ms.

On this poster we explore this surprising ability in a model of the honeybee antennal lobe. We hypothesize that a winner-take-all inhibitory network (see Figure 1) of local neurons (LNs) in the antennal lobe has a symmetry-breaking effect, such that the response pattern in projection neurons (PNs) to an asynchronous mixture is different from the response pattern to the corresponding synchronous mixture for an extended period of time beyond the initial odor onset where the two mixture conditions actually differ. The prolonged difference between response patterns to synchronous and asynchronous mixtures could facilitate odor-background segregation in downstream circuits of the olfactory pathway.

**Figure 1:**
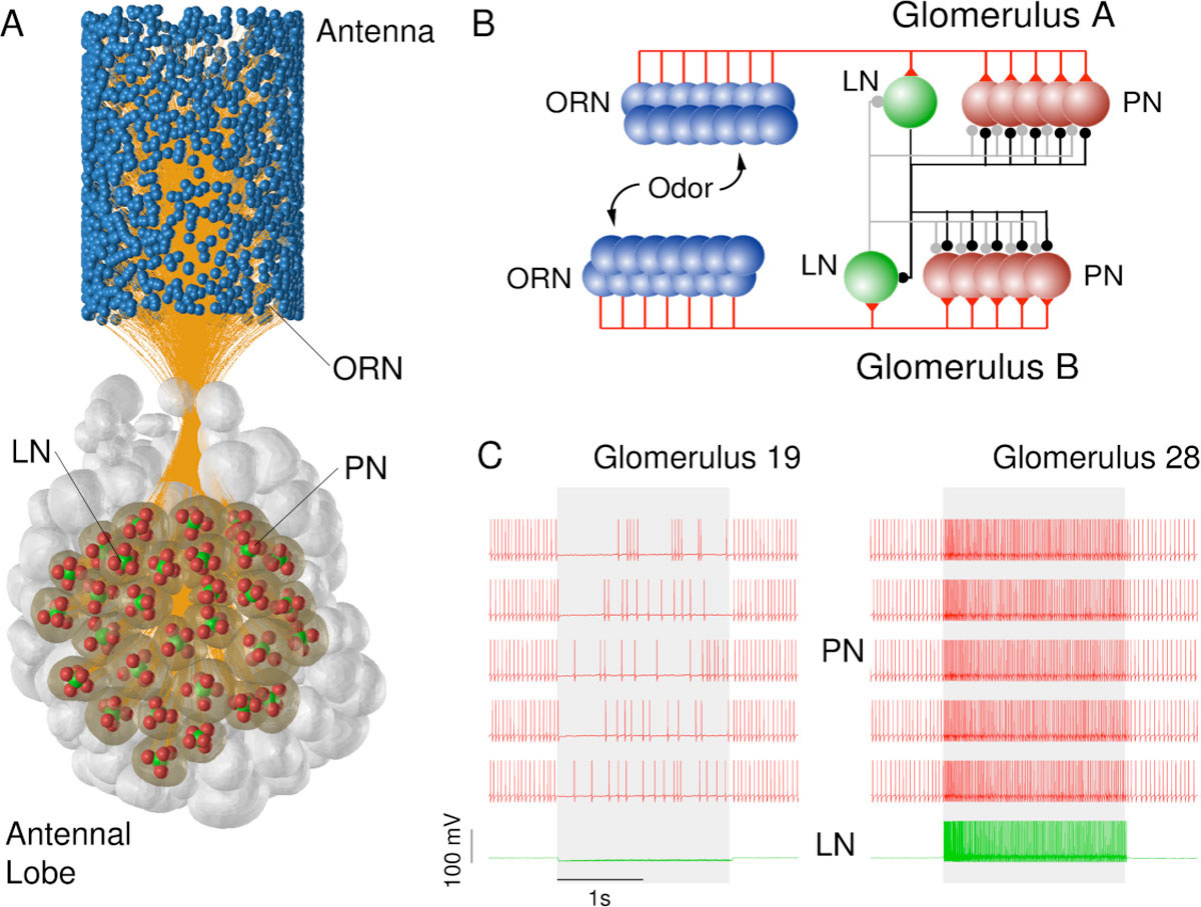
**A Anatomy of the olfactory system of the honeybee**. Olfactory receptor neurons (ORNs) on the antenna are depicted as blue spheres, the glomeruli of the AL were derived from the 3D AL atlas [3] and each glomerulus for which we had sufficient data (transparent) was modeled with 5 PNs (red spheres) and one LN (green spheres). **B **Functional network architecture. ORNs of the same type converge onto the same glomerulus in which they excite all PNs and the LN. The LN of each glomerulus inhibits all PNs of the same glomerulus and of other glomeruli. The strength of inhibition was chosen proportional to the correlation of PN activity of the glomeruli as observed in the data. The LNs also inhibit each other, forming a winner-take-all circuit. **C **example simulation data from PNs (red) and LNs (green).

We present a detailed data-driven model of the bee antennal lobe that reproduces a large data set of experimentally observed odor responses [2] and demonstrate with this model that our hypothesis is consistent with the current knowledge of the olfactory circuits in the bee brain.

## References

[B1] Szyszka P Stierle JS Biergans S Galizia CG The speed of smell: odor-object segregation within milliseconds PLoS One 2012 7 e36096 10.1371/journal.pone.0036096 22558344 PMC3338635

[B2] Ditzen M Odor concentration and identity coding in the antennal lobe of the honeybee Apis mellifera 2005 Ph.D. thesis. Freie Universität Berlin. Berlin

[B3] Galizia CG McIlwrath SL Menzel R A digital 3D atlas of the honeybee antennal lobe based on optical sections acquired using confocal micoscropy Cell Tissue Res 1999 295 383 394 10.1007/s004410051245 10022959

